# The Relation of Noise to Good Health

**Published:** 1893-07

**Authors:** 


BUFFALO MEDICAL AND SURGICAL JOURNAL
A MONTHLY REVIEW OF MEDICINE AND SURGERY.

*EDITORS:*
THOMAS LOTHROP, M. D. - - WM. WARREN POTTER, M. D.

All communications, whether of a literary or business character, should be addressed to the managing editor: 284 FRANKLIN STREET, BUFFALO, N. Y.

VOL. XXXII. JULY, 1893. No. 12.

### THE RELATION OF NOISE TO GOOD HEALTH.

SOME of the lay newspapers are discussing the question of noise as related to the peace and comfort of the people resident in large cities. Such a discussion is timely, and deserves to be carried on until reform has been obtained in this matter of great abuse. There is not the slightest doubt but that the noise, bustle, and confusion, incident to the conduct of traffic in large cities, has much to do with nerve perturbation and disease, either as an exhaustive factor or as an aggravating contributory element. We refer now not to that essential noise which belongs to the proper conduct of business affairs, but to a superfluous group of noises that could be prevented by a little care and the exercise of prudence and good judgment.

The shouting of wares through the streets should be prohibited as a noise that is especially disagreeable and harmful in its character. Lawn mowers should be used at a seasonable hour, so that neighbors who are ill, or who must sleep during the early morning hours, need not be disturbed thereby. The ringing of church bells should be entirely prohibited by municipal law as a noise that is worse than disagreeable, being a positive nuisance, besides being totally unnecessary. Parents should be careful in the selection of toys for their children, that they do not become also a noise-nuisance instead of a delight to the community and neighborhood. Trumpets, drums, whistles, and other noisy trinkets should be carefully eliminated from the repertory of children amusements. Steam-whistles also are quite unnecessary, and their screeching noise is of the worst nature. There is a long list of useless noises that might be eradicated without laying a stumbling block in the path of legitimate traffic, progress, or money-getting. We have only hinted at a few of them, but, should occasion offer, we may refer to the subject at another time more in detail. Our object has been to throw out a hint that would open up a channel of thought to our readers, who are naturally the leaders of reform on all subjects that pertain to health and prosperity and in municipal sanitation.

The 130 or more miles of asphalt pavement that Buffalo possesses, go a long way towards reducing the rattling sounds of vehicles in the conduct of traffic, as well as in the pleasure-riding of our citizens. Let us all, on every proper occasion, preach the doctrine of less noise in our city and stronger nerves for our citizens.

